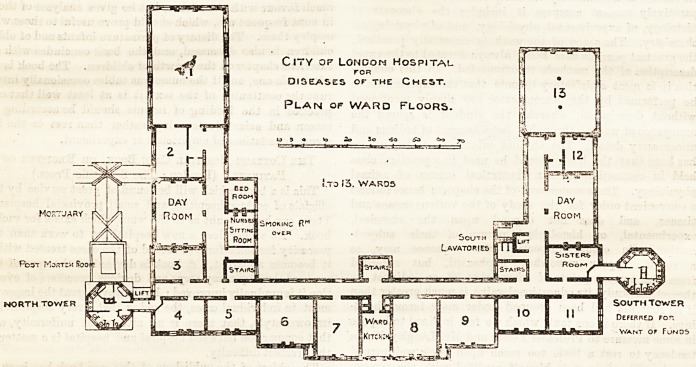# Hospital Construction

**Published:** 1897-02-27

**Authors:** 


### The Institutional Workshop.

### HOSPITAL CONSTRUCTION.

### CITY OF LONDON HOSPITAL FOR DISEASES OF THE CHEST.

The accompanying plan of the first floor indicates the improved sanitary arrangements executed and proposed at the above institution. As is here shown, the whole of the baths, lavatories, slop sinks, and w.c.'s on this and on each of five floors, have been accommodated in a north tower, properly disconnected by a cross-ventilated lobby or bridge, from the remainder of the building, and only want of funds prevents the completion of a similar project at the southern end of the building. A mortuary and post-mortem room have been provided above ground in lieu of the rooms formerly used for these purposes in the basement of the building, and the drainage has been greatly improved. It is pointed out that the plans of this institution, as given in Burdett's "Hospitals and Asylums of the World," do not include these alterations, and are so far misleading, inasmuch as the alterations were completed at the date which appears on the title page of that work. It gives us great pleasure to supplement the information contained in that work by giving publicity to a much-needed amendment in this excellent institution, upon the accomplishment of which the committee of management is to be heartily congratulated.

## Figures and Tables

**Figure f1:**